# ﻿The tiniest violin: the male of *Loxoscelesvallenar* (Araneae, Sicariidae)

**DOI:** 10.3897/zookeys.1206.122469

**Published:** 2024-07-11

**Authors:** Ivan L. F. Magalhaes, Hernán A. Iuri, Antonio D. Brescovit, Jaime Pizarro-Araya

**Affiliations:** 1 División Aracnología, Museo Argentino de Ciencias Naturales “Bernardino Rivadavia” — CONICET, Av. Angel Gallardo 470, C1405DJR, Buenos Aires, Argentina División Aracnología, Museo Argentino de Ciencias Naturales “Bernardino Rivadavia” — CONICET Buenos Aires Argentina; 2 Laboratório de Coleções Zoológicas, Instituto Butantan, Av. Vital Brasil, 1500, 05503-900, São Paulo, SP, Brazil Instituto Butantan São Paulo Brazil; 3 Laboratorio de Entomología Ecológica (LEULS), Departamento de Biología, Facultad de Ciencias, Universidad de La Serena, Casilla 554, La Serena, Chile Universidad de La Serena La Serena Chile; 4 Programa de Doctorado en Conservación y Gestión de la Biodiversidad, Facultad de Ciencias, Universidad Santo Tomás, Ejército 146, Santiago, Chile Universidad Santo Tomás Santiago Chile; 5 Instituto de Ecología y Biodiversidad (IEB), Ñuñoa, Santiago, Chile Instituto de Ecología y Biodiversidad (IEB) Santiago Chile; 6 Grupo de Artrópodos, Sistema Integrado de Monitoreo y Evaluación de Ecosistemas Forestales Nativos (SIMEF), Santiago, Chile Grupo de Artrópodos, Sistema Integrado de Monitoreo y Evaluación de Ecosistemas Forestales Nativos (SIMEF) Santiago Chile

**Keywords:** Atacama, brown recluse spider, Chile, desert, Matorral, sexual dimorphism, taxonomy, violin spider

## Abstract

In recent years, several endemic species of *Loxosceles*, violin spiders, have been described from the North-Central Chile biodiversity hotspot, some of which have ambiguous placement within the species groups of the genus. In a recent expedition to the Atacama region, we collected male specimens representing new records of two recently described species: *Loxoscelesvicentei* Taucare-Ríos, Brescovit & Villablanca, 2022 and *Loxoscelesvallenar* Brescovit, Taucare-Ríos, Magalhaes & Santos, 2017 (Araneae, Sicariidae). Males of the latter are hitherto unknown and are here described for the first time. Examination of the morphology of these species revealed characters such as an embolic keel and digitiform median receptacles, which suggest they do not belong in the *laeta* species group, but rather in the *spadicea* species group, which is briefly re-diagnosed. With carapace lengths smaller than 2 mm, the newly discovered males of *L.vallenar* are the tiniest members of the genus. In addition, males of this species bear strong macrosetae in the clypeus, a sexually dimorphic character not previously reported in *Loxosceles*.

## ﻿Introduction

Central Chile is renowned as a biodiversity hotspot (Myers et al. 2000; Mittermeier et al. 2011). However, there are knowledge gaps for its arthropod species, specifically referring to Linnean and Wallacean shortfalls, resulting in challenges for regional conservation (Vergara-Asenjo et al. 2023). Regarding spiders, numerous new species have been described in the last decade, partially tackling such shortfalls (e.g., Laborda et al. 2013; Bustamante et al. 2014; Brescovit and Sánchez-Ruiz 2016; Grismado and Pizarro-Araya 2016; Brescovit et al. 2017; Griotti et al. 2022; Taucare-Ríos et al. 2022). In particular, the spider genus *Loxosceles* includes five species endemic to Central Chile: *L.coquimbo* Gertsch, 1967, *L.diaguita* Brescovit, Taucare-Ríos, Magalhaes & Santos, 2017, *L.pallalla* Brescovit, Taucare-Ríos, Magalhaes & Santos, 2017, *L.vallenar* Brescovit, Taucare-Ríos, Magalhaes & Santos, 2017 and *L.vicentei* Taucare-Ríos, Brescovit & Villablanca, 2022. Four of these have been described in the last decade, indicating that the diversity of this medically important genus has been previously underestimated in Chile.

*Loxosceles* violin spiders include 149 species occurring naturally mainly in Africa, the Americas and the Mediterranean region (WSC 2024). The genus dates back to the Cretaceous (Binford et al. 2008; Magalhaes et al. 2019), and due to its high morphological and taxonomic diversity, it has been separated into several species groups (Gertsch 1967), most of which have resisted the scrutiny of phylogenetic analyses (Binford et al. 2008). Three of the Central Chile endemics have been placed in the *laeta* species group – *L.coquimbo*, *L.vallenar* and *L.vicentei* – and thus were assumed to be closely related to *Loxosceleslaeta* (Nicolet, 1849), a species that may cause serious injury in humans due to the dermonecrotic activity of its venom (Schenone et al. 1989). The other two species have been placed tentatively in the *spadicea* species group (*L.diaguita*) or left unplaced due to its aberrant genital morphology (*L.pallalla*) (see Brescovit et al. 2017). This seems to indicate that the *laeta* species group is the most diverse among Chilean species.

**Figure 1.**
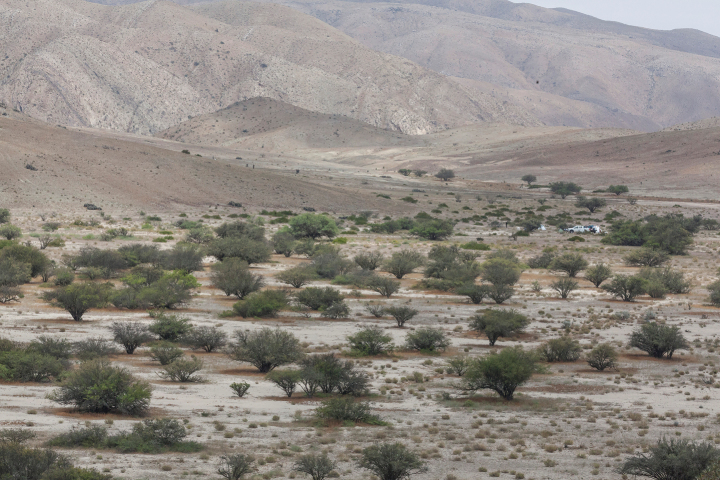
Habitat in Boquerón Chañar, Atacama, where *Loxoscelesvallenar* and *L.vicentei* were collected.

**Figure 2.**
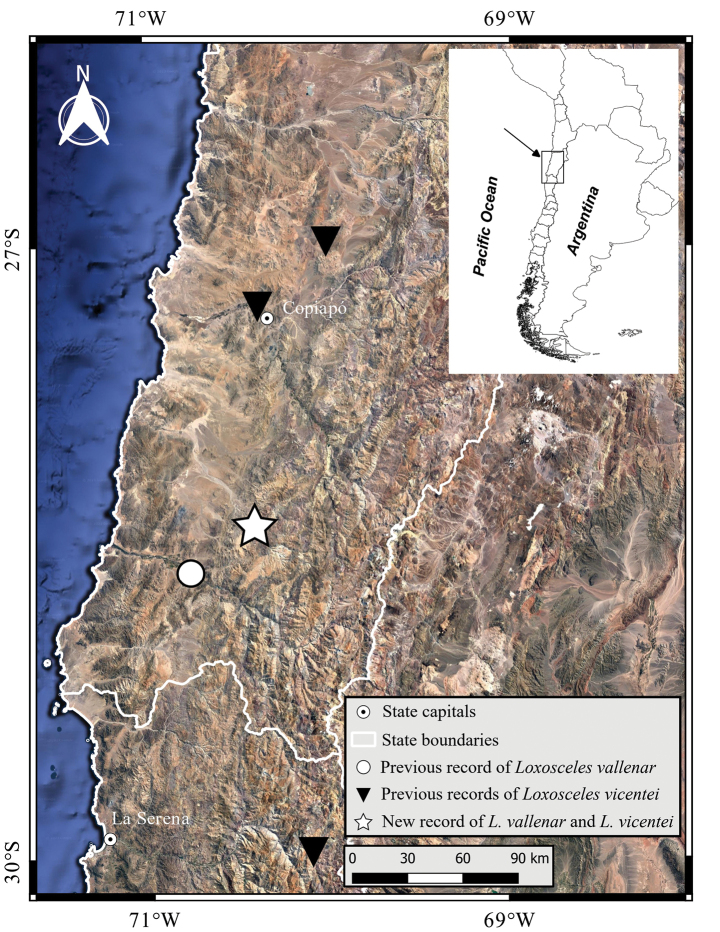
Distribution of *Loxoscelesvallenar* and *Loxoscelesvicentei*. The star represents a new record for both species in the same locality.

**Figure 3.**
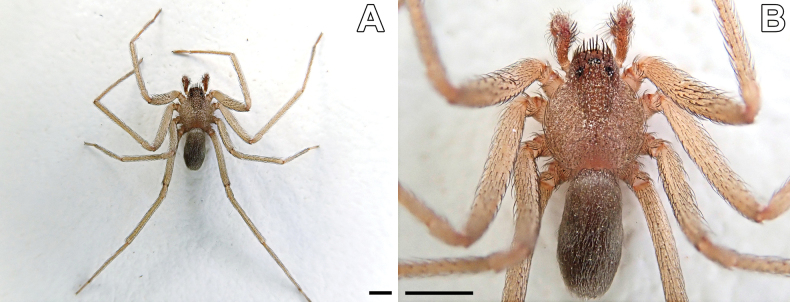
*Loxoscelesvallenar*, live male specimen from Boquerón Chañar **A** entire specimen **B** close-up. Notice the clypeal setae. Scale bar: 1 mm.

**Figure 4.**
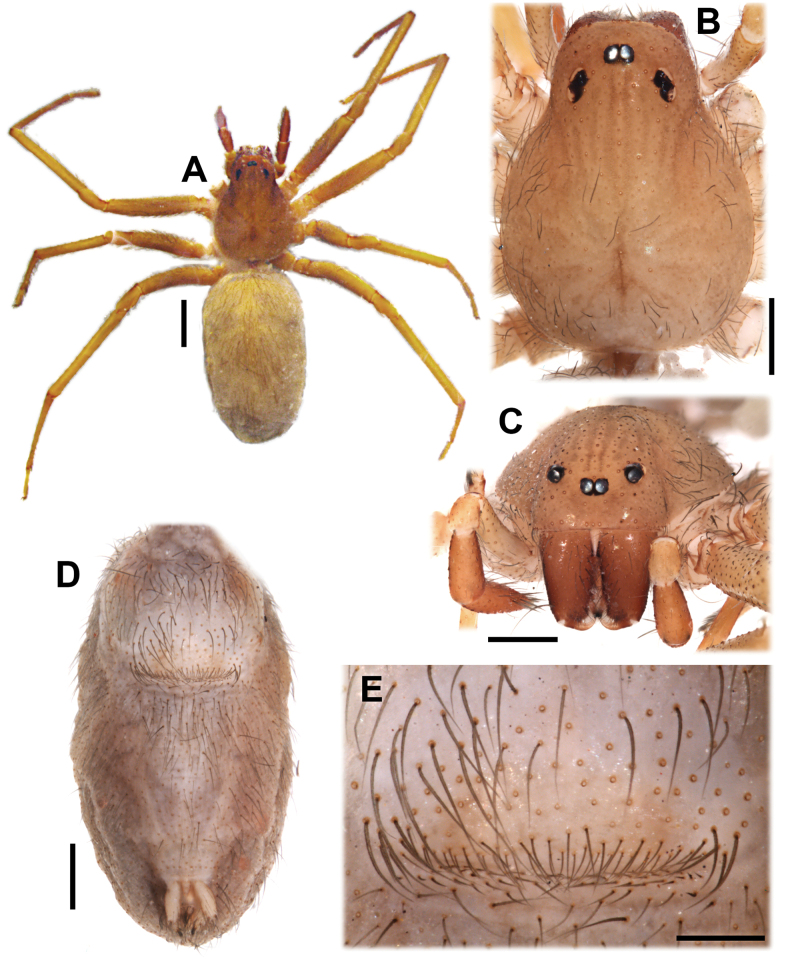
*Loxoscelesvallenar* Brescovit, Taucare-Ríos, Magalhaes & Santos, females **A** holotype from Vallenar (AMNH), dorsal **B–E** female from Boquerón Chañar (MACN-Ar 44131) **B** dorsal **C** carapace, anterior **D** abdomen, ventral **E** genital region, ventral. Scale bars: 1000 µm (**A**); 500 µm (**B–D**); 200 µm (**E**).

In a recent expedition to Boquerón Chañar (Fig. 1), an area in the Atacama region, Chile (Fig. 2), we collected males of *Loxosceles* that did not fit with any described specimens in the literature, mainly because of the modified clypeal macrosetae (Fig. 3). In the same area, we found a similar-sized female specimen of *Loxoscelesvallenar* (Fig. 4B–E), a species hitherto known only by the females, which led us to conclude that the undescribed males are conspecific with them. In addition to describing this male, the new specimens prompted us to re-evaluate the placement of Chilean *Loxosceles* in the different species groups, as the newly discovered specimen does not present characters consistent with the *laeta* species group. Finally, we report sexually dimorphic characters that had not been previously described in *Loxosceles*.

## ﻿Material and methods

### ﻿Fieldwork

Specimen collection was performed during November 2023 (late spring) in several places of Quebrada Algarrobal, near Boquerón Chañar, in the Atacama region. The specimens were manually collected by searching on the ground at night using a headlight. Photographs of the live specimens were taken with an Olympus Tough TG-5 digital camera.

### ﻿Microscopy

Endogynes were examined in lactic acid after digestion using pancreatin solution (Álvarez-Padilla and Hormiga 2008). Images of the holotype were taken with a Leica DM4000B Microscope and a Leica M205C stereomicroscope at Instituto Butantan, São Paulo. For other specimens, images in multiple focal planes were taken with a Leica M205C stereomicroscope or an Olympus BH2 compound microscope in Museo Argentino de Ciencias Naturales Bernardino Rivadavia (MACN), Buenos Aires, and then combined with Helicon Focus 7 (HeliconSoft, Kharkiv, Ukraine); a camera lucida was used to make a schematic drawing. Material for scanning electron microscopy was dried in a series of ethanol solutions of increasing concentrations, with a final step of reagent-grade, pure hexamethyldisilazane (Sigma-Aldrich), and then air-dried. Samples were mounted on aluminum stubs and sputter coated with gold-palladium, and then examined under a high vacuum with a Zeiss GeminiSEM 360 scanning electron microscope in MACN.

### ﻿Description

The format of the description follows Brescovit et al. (2017), with slight modifications; leg measurements are given as total length (femur, patella, tibia, metatarsus and tarsus lengths). Descriptions and lists of examined material were prepared using automated spreadsheets (Magalhaes 2019). Specimens whose geographical coordinates are placed between square brackets were georeferenced by us using Google Earth (Google, Mountain View, USA); coordinates placed between parentheses represent those on the original labels. Geographic coordinates of the collecting sites were recorded using a GPS Garmin eTrex, Vista C. The distribution map was generated using QGIS 3.22.10-Białowieża (distributed under the GNU General Public License, www.gnu.org/licenses) using Land-Sat satellite images (Chávez 1996; Chander and Markham 2003) and supporting cartography from the Instituto Geográfico Militar at a scale of 1:250,000 (IGM 1986).

### ﻿Collections

The material studied in this paper is deposited in the American Museum of Natural History, New York, U.S.A. (AMNH; curator L. Prendini) and in the Museo Argentino de Ciencias Naturales “Bernardino Rivadavia”, Buenos Aires, Argentina (MACN-Ar; curator M.J. Ramírez).

## ﻿Taxonomy

### ﻿Family Sicariidae Keyserling, 1880

***Loxosceles* Heineken & Lowe, 1832**

**The *spadicea* species group**

**Figure 5.**
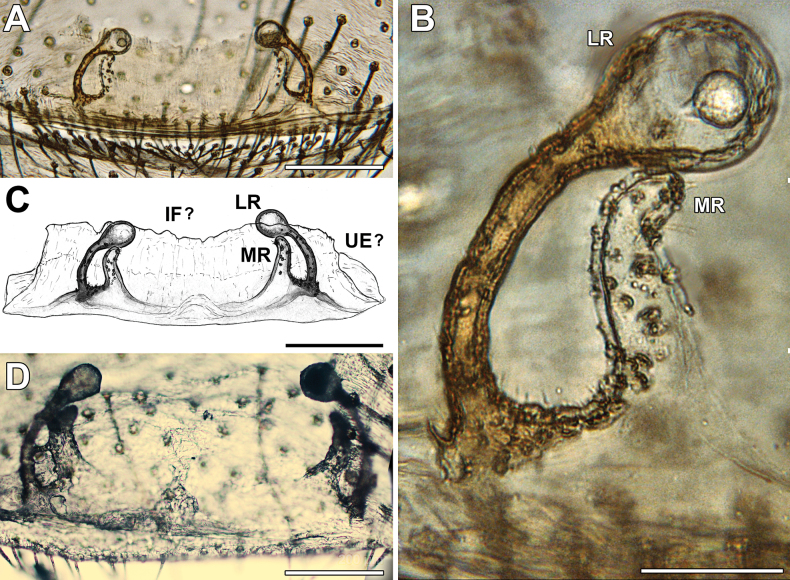
*Loxoscelesvallenar*, median and lateral receptacles of the female genitalia **A–C** female from Boquerón Chañar (MACN-Ar 44131) **D** holotype from Vallenar (AMNH). Abbreviations: IF? = putative interpulmonary fold, LR = lateral receptacle, MR = median receptacle, UE? = putative uterus externus; details could not be resolved in light microscopy. Scale bars: 200 µm (**A, C, D**); 50 µm (**B**).

**Figure 6.**
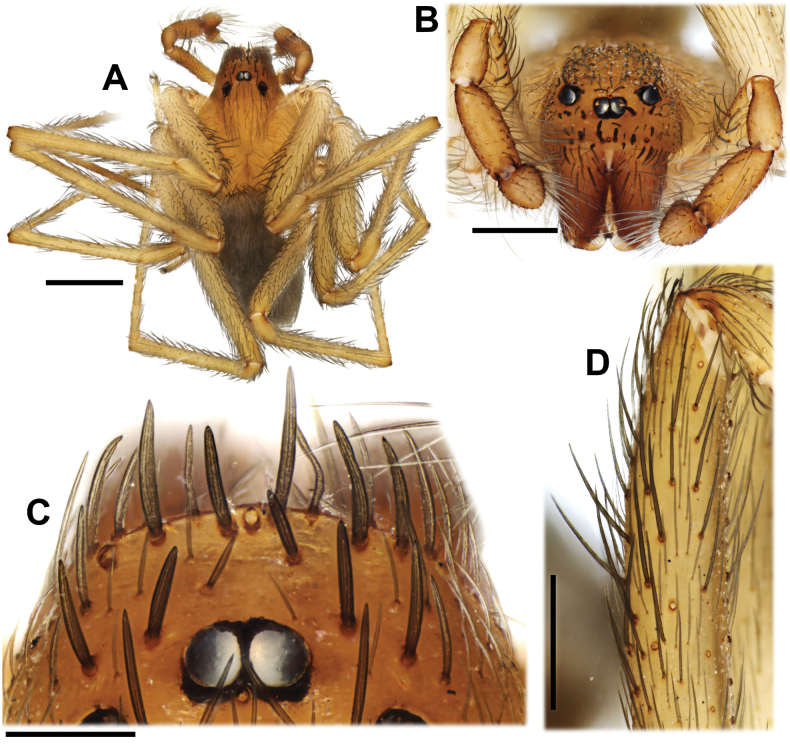
*Loxoscelesvallenar*, male from Boquerón Chañar (MACN-Ar 44130) **A** habitus, dorsal **B** clypeus, anterior **C** clypeus, dorsal **D** left femur I, prolateral. Scale bars: 1000 µm (**A**); 500 µm (**B, D**); 200 µm (**C**).

**Figure 7.**
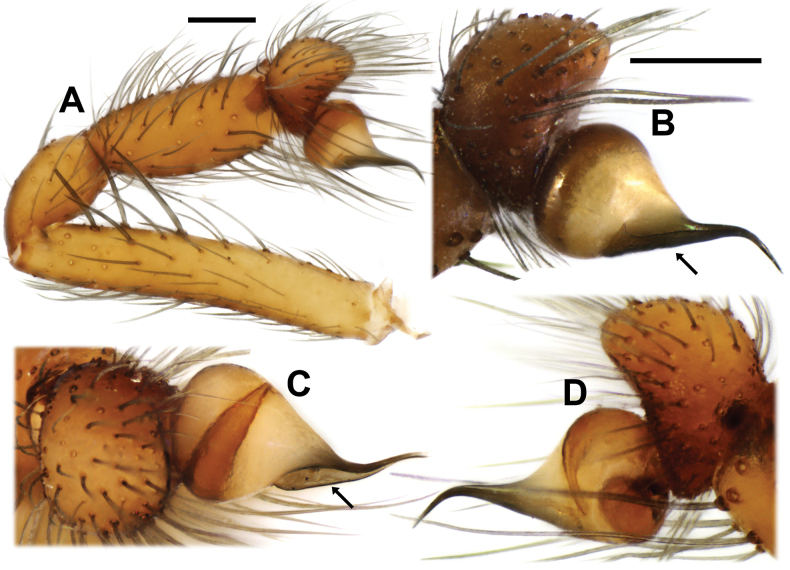
*Loxoscelesvallenar*, males from Boquerón Chañar (MACN-Ar 44130) (except B, MACN-Ar 44129, dry specimen before sputter-coating), left palps **A, B** prolateral **C** dorsal **D** retrolateral. Arrows indicate embolic keel. Scale bars: 200 µm (**B–D** to the same scale).

**Figure 8.**
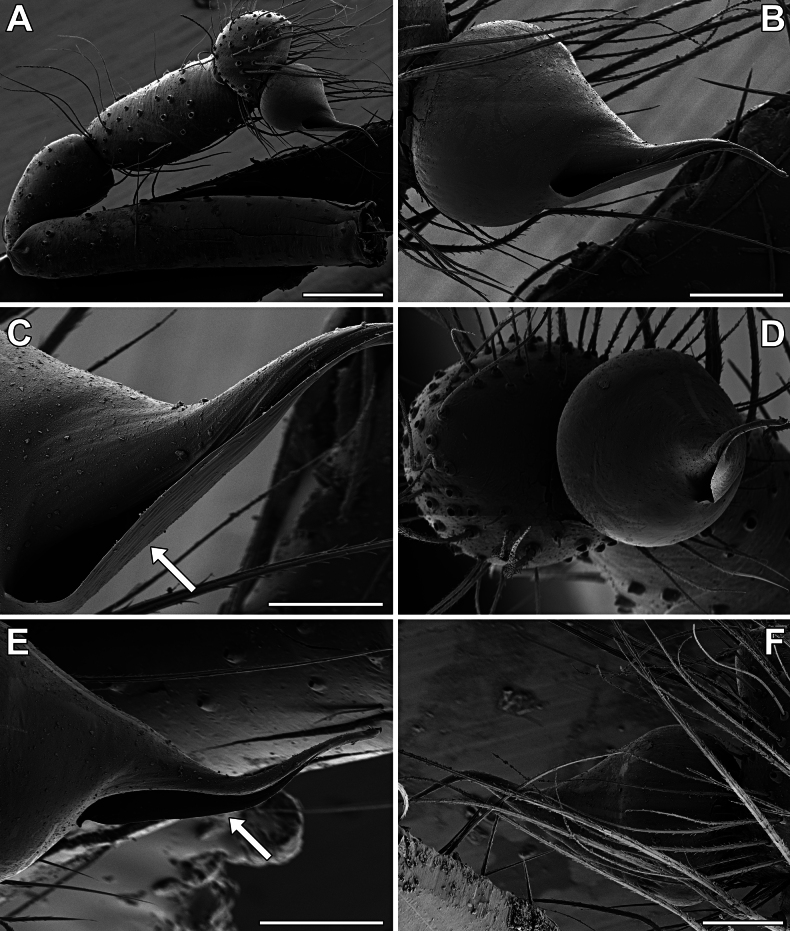
*Loxoscelesvallenar*, male left palp (MACN-Ar 44129) under scanning electron microscopy **A** prolateral **B** bulb, prolateral **C** detail of embolic keel **D** bulb, apical **E** bulb, dorsal **F** bulb, retrolateral. Arrows indicate embolic keel. Scale bars: 200 µm (**A**); 100 µm (**B, E, F**); 50 µm (**C**).

**Figure 9.**
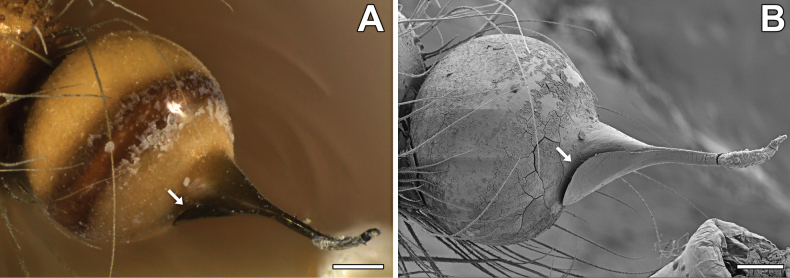
*Loxoscelesvicentei* Taucare-Ríos, Brescovit & Villablanca, 2022, male from Boquerón Chañar (MACN-Ar 44128), left bulb, prolateral, in light (**A**) and scanning electron microscopy (**B**). Arrows indicate embolic keel. Scale bars: 100 µm.

**Diagnosis.** Small to medium-sized *Loxosceles* (body length 2–8 mm). The carapace may be uniform brown or orange (e.g., *L.vallenar*, *L.hirsuta*, *L.diaguita*; Fig. 4B) or bear distinctive markings (e.g., *L.vicentei*; Taucare-Ríos et al. 2022, fig. 1), and may be distinctly hirsute. The leg formula is usually 2413, but may be 2143 in some females, and 4213 in some species (*L.vicentei* males and females, *L.vallenar* females). The most distinctive features of the group are in the genitalia: the female genitalia have the receptacles separated by two to several times their width at the base, and the receptacles are short and digitiform, usually bearing a small head (Fig. 5); the uterus externus and the interpulmonary fold are not particularly sclerotized or modified. Male palps have the cymbium short and diamond-shaped (Fig. 6B); the bulb is always globose at the base, and the embolus always bears a keel (“wing or carina” sensu Gertsch 1967) (Figs 7–9, arrows); the embolus may be quite short (Fig. 7) to quite long (Brescovit et al. 2017, fig. 7). The first metatarsi of males are unmodified.

**Species included.** So far, the group encompasses *L.hirsuta* Mello-Leitão, *L.intermedia* Mello-Leitão, *L.spadicea* Simon, *L.anomala* Mello-Leitão and *L.diaguita*. We argue that, based on the diagnosis above, the following species also belong in the group: *L.vallenar*, *L.coquimbo*, *L.vicentei*, and *L.pallalla*.

#### ﻿ Loxosceles vallenar

Taxon classificationAnimaliaAraneaeSicariidae

Brescovit, Taucare-Ríos, Magalhaes & Santos, 2017

F5EB1B9D-A773-59CB-A77D-889AD28B4D25

Figs 3 , 4 , 5 , 6 , 7 , 8

Loxosceles vallenar Brescovit, Taucare-Ríos, Magalhaes & Santos, 2017: 14, fig. 8E.

##### Holotype (examined).

Chile • 1 ♀; Atacama, Prov. Huasco, Vallenar, 3 km S Vallenar; 460 m.a.s.l. [28.601°S, 70.77°W]; 7 Jan. 1985; N Platnick, O Francke leg.; deposited in AMNH.

##### New records.

Chile • 1 ♂; Atacama, Prov. Huasco, Boquerón Chañar; Algarrobal; 992 m.a.s.l.; (28.3708°S, 70.4128°W); 24–25 Nov. 2022; J Pizarro-Araya, FM Alfaro, JE Barriga, AA Ojanguren-Affilastro, HA Iuri & JE Calderón leg.; IFM-2580; MACN-Ar 44129 • 1 ♂; same collecting data; MACN-Ar 44130 • 1 ♀; same collecting data; MACN-Ar 44131.

##### Remarks.

The genitalia of the holotype was incorrectly illustrated by Brescovit et al. (2017), as the digitiform, membranous median receptacles were overlooked. Re-examination of the holotype revealed they are present (Fig. 5D). We briefly describe the females collected with the males, especially their endogyne, which is in a better state than that of the holotype. The hitherto undescribed male has a palpal bulb bearing an embolic keel and overall morphology concordant with the *spadicea* species group (Figs 6–8). Thus, the species is moved from the *laeta* species group to the *spadicea* species group.

##### Diagnosis.

Males resemble those of other members of the *spadicea* species group by the subtriangular cymbium in dorsal view (Fig. 6B) and embolus bearing a keel (Fig. 7C), but can be distinguished by the clypeus bearing macrosetae (Figs 3, 6A–C) (vs. clypeus without macrosetae) and by the more piriform, tapering copulatory bulb (Fig. 7B) (vs. bulb clearly globose; Gertsch 1967, figs 1, 4, 9). Females resemble those of other members of the *spadicea* species group by the short and digitiform median receptacle, but differ by the inwards curved base of the lateral receptacle (Fig. 5) (vs. base of the lateral receptacle sinuous to convoluted; Gertsch 1967, figs 11–14).

##### Description.

Male from Boquerón Chañar, Atacama, Chile (MACN-Ar 44130). ***Coloration in ethanol*** (Fig. 6A). Carapace yellowish-brown with slightly darker pars cephalica, eyes surrounded by black pigment rings. Chelicerae orange brown. Labium light brown. Endites brownish-cream at base, darker at median third, whitish at tip. Palps yellowish-brown, tarsus darkest. Sternum brownish-cream. Legs uniformly light brown. Opisthosoma uniformly brownish-gray. ***Measurements***. Total length 3.62. Carapace length 1.92, width 1.48. Clypeus height 0.23. Eye diameters and interdistances: ALE 0.11, PLE 0.12, PME 0.10, ALE–PME 0.13. Sternum length 1.13, width 0.95. Palp: femur length 1.08, height 0.18, tibia length 0.60, height 0.25, tarsus length 0.27. Leg I 8.6 (2.38, 0.58, 2.43, 2.38, 0.83). Leg II 10.11 (2.82, 0.59, 3.03, 2.84, 0.83). Leg III 7.53 (2.23, 0.57, 1.95, 2.14, 0.64). Leg IV 9.88 (2.82, 0.58, 2.75, 2.96, 0.77). Leg formula 2413. Abdomen: length 1.67, width 1.08. Femur I with ~17 prolateral macrosetae in a subdistal patch (Fig. 6D). Metatarsus I unmodified. Clypeus with ~20 macrosetae (Fig. 6C). ***Male genitalia*** (Figs 7, 8). Palpal femur with 5 prolateral macrosetae in apical third, tibia slightly swollen, with two pronounced condyles apically, cymbium short and subtriangular in dorsal view, bulb short and rounded, embolus curved and tapering retrolaterally, with a prolateral keel, without micro-spines (Fig. 8). State of the specimen: good; left palp dissected.

Female from Boquerón Chañar, Atacama, Chile (MACN-Ar 44131). Coloration and general structure as in the holotype (Fig. 4). Carapace length 2.20, width 1.68. Genital region externally pubescent but without stronger sclerotization (Fig. 4E). ***Endogyne*** (Fig. 5). Median receptacle digitiform, slightly sinuous and lightly sclerotized, bearing glandular pores throughout. Lateral receptacle with thin, sclerotized and arched base bearing glandular pores, leading to a rounded, unsclerotized head lacking pores; fold (presumably uterus externus and/or interpulmonary fold) short and membranous. State of the specimen: good; endogyne dissected.

##### Variation.

The two males and two females examined have seemingly identical genitalia. Two males: total length 3.62 to 3.74; carapace length 1.92 to 1.97; tibia I length 2.43 to 2.73. Both females have a carapace length of 2.2 and vary in total length from 5.53 to 6.00.

##### Natural history.

The label data indicates that the holotype was collected “in scrubby mountain-side, under rocks”. The specimens from Boquerón Chañar were collected at night, searching with headlights. The male specimens were actively walking on the ground.

##### Habitat.

Specimens of *Loxoscelesvallenar* and *L.vicentei* were collected in vegetation zones characterized by an inland Mediterranean-desert shrubland of *Adesmiaargentea* Meyen and *Bulnesiachilensis* Gay (Luebert and Pliscoff 2006). These areas are defined by an extremely open shrubland with tall shrubs and the presence of tree species. Shrubs include *A.argentea*, *Bulnesiachilensis*, *Balsamocarponbrevifolium* Clos, *Cordiadecandra* Hook. & Arn., *Heliotropiumsinuatum* (Miers) I.M. Johnst., *Pintoachilensis* Gay, and *Proustiailicifolia* Hook. & Arn. Additionally, low shrubs such as *Caesalpiniaangulata* (Hook. & Arn.) Baill., *Enceliacanescens* Lam., *Pleurophorapungens* D. Don, and cacti like *Cumulopuntiasphaerica* (C.F. Först.) E.F. Anderson and *Trichocereuscoquimbanus* (Molina) Britton and Rose are common. Herbaceous plants abound during the rainy season, including species like *Cruckshanksiapumila* Clos and *Argyliairradian* (L.) D. Don. This entire environment is dominated by trees such as *Neltumachilensis* (Molina) Hughes & Lewis, *Geoffroeadecorticans* (Gillies ex Hook. & Arn.) Burkart, *Acaciacaven* (Molina) Molina, and *Schinuspolygama* (Cav.) Cabrera (Luebert and Pliscoff 2006) (Fig. 1). The climate in the area is semiarid subtropical Mediterranean in the northern margin and marine subtropical Mediterranean in the southern margin (Novoa and Villaseca 1989). The total precipitation recorded in the study area (Vallenar Station, 28°33'6.11"S, 70°47'25.92"W, 421 m.a.s.l.) in 2022 was 83.8 mm and was concentrated in July (66.5 mm) and June (4.9 mm) (CEAZA-Met 2024).

##### Distribution.

Originally described from Vallenar, the new record extends the distribution of the species about 40 km northeastward. Both points are in Huasco Province, Atacama region, Chile (Fig. 2).

#### ﻿ Loxosceles vicentei

Taxon classificationAnimaliaAraneaeSicariidae

Taucare-Ríos, Brescovit & Villablanca, 2022

EE72F9D6-971C-5343-A346-7043F9089494

Fig. 9

Loxosceles vicentei Taucare-Ríos, Brescovit & Villablanca, 2022: 158, figs 1A–B, 2A–D, 4A–B.

##### Holotype (not examined).

Chile • 1 ♂; Coquimbo, Elqui Prov., Vicuña, Fondo El Calvario, near Juntas del Toro, 29°58'30.97"S, 70°6'11.86"W, 2050 m.a.s.l., 14 Oct. 2021, V Villablanca Miranda, J Villablanca Rivera & A Taucare-Ríos leg.; Museo Nacional de Historia Natural, Santiago, Chile, MNNC 8371.

##### New record.

Chile • 1 ♂; Atacama, Prov. Huasco, Boquerón Chañar; Algarrobal; 992 m.a.s.l., (28.3708°S, 70.4128°W); 24–25 Nov. 2022; J Pizarro-Araya, FM Alfaro, JE Barriga, AA Ojanguren-Affilastro, HA Iuri & JE Calderón leg.; IFM-2579; MACN-Ar 44128.

##### Remarks.

The new record is about 183 km north of the type locality of *L.vicentei*, but within the distribution of this species presented by Taucare-Ríos et al. (2022). The embolic keel we observed in our specimens (Fig. 9, arrows) was not mentioned in the original description of the species but can nonetheless be seen in their figures (Taucare-Ríos et al. 2022, fig. 2A). The holotype has a slightly longer embolus and a seemingly larger keel; a larger series of specimens must be examined to evaluate the significance of these morphological differences. The presence of an embolic keel, allied with the digitiform median receptacles of the female (Taucare-Ríos et al. 2022, fig. 2D), hints that *L.vicentei* belongs in the *spadicea* species group, rather than in the *laeta* species group. Taucare-Ríos et al. (2022) present a map with four records, but their list of material examined indicated only three localities; the fourth record had been included by mistake (A. Taucare-Ríos, in litt.), and thus, we omit it in our map.

##### Habitat.

See remarks under *L.vallenar* above.

## ﻿Discussion

The newly described male indicates that *Loxoscelesvallenar* is the smallest species among violin spiders. With a carapace length between 1.92 and 1.97, males are smaller than those of *Loxoscelesdejagerae* Lotz, 2017 from South Africa (carapace lengths of 2.0–3.2; see Lotz 2017: 478), the previously smallest representative. Some other members of the *spadicea* group are also small, such as *L.spadicea* and *L.hirsuta* (carapace length 2.5; see Gertsch 1967).

The male of *Loxoscelesvallenar* presents a secondary sexual character that has not been previously reported in the genus: a group of strong macrosetae in the clypeus. Secondary sexual characters in *Loxosceles* males are rare and usually associated with the legs, such as sinuous tibiae or metatarsi (see Brescovit et al. 2017; Bertani et al. 2018) or the presence of sexually dimorphic macrosetae patches (Bertani et al. 2018; Magalhaes et al. 2022). *Loxoscelesvallenar* males also present a prolateral patch of macrosetae in the first femur. Presumably, some of these modifications of the legs may serve as clasping spurs during mating; for instance, *L.laeta* males grab the female coxae with their first legs during courtship and mating (Fischer 2007). This behavior is absent in *L.hirsuta* and *L.intermedia* (Fischer and Vasconellos-Neto 2000; Fischer and Silva 2001), whose males have unmodified legs. In these two species, however, males and females touch pedipalps during courtship, a very unusual behavior; we wonder if their embolic keel (see Gertsch 1967, fig. 3) plays a role in this. Regarding the clypeal macrosetae, several other Synspermiata (the clade where Sicariidae belongs) have males with modified clypei, such as *Relictocera* Li & Li (Psilodercidae), *Unicorn* Platnick & Brescovit (Oonopidae) and *Perania* Thorell (Pacullidae) (Lehtinen 1981; Platnick and Brescovit 1995; Chang et al. 2019). In most Synspermiata, the male faces the underside of the female during palpal insertions (see Fischer 2007). In this position, such clypeal modifications may help locking or stimulating the underside of the female. It remains to be tested if this is the case in *L.vallenar*.

We argue that *L.vallenar* and *L.vicentei* belong in the *spadicea* species group rather than in the *laeta* species group. Additionally, we re-examined the male of *Loxoscelescoquimbo* described by Brescovit et al. (2017), revealing that its palpal bulb also bears an embolic keel, albeit small (Brescovit et al. 2017, fig. 2B). Together with the similar structure of the endogyne of *L.coquimbo* and *L.vicentei*, this suggests that the former also belongs in the *spadicea* species group. This indicates that the *spadicea* species group has diversified on both sides of the Andes: in Chile, it includes *L.diaguita*, *L.vallenar*, *L.coquimbo*, *L.vicentei*, and *L.pallalla*, which also possesses an embolic keel. East of the Andes, it includes *L.hirsuta*, *L.intermedia*, *L.spadicea* and *L.anomala*. The *laeta* group is thus represented in Chile only by two species: *L.surca* Gertsch, which occurs in Andean areas in the north of the country, and the synanthropic *L.laeta*.

## Supplementary Material

XML Treatment for Loxosceles vallenar

XML Treatment for Loxosceles vicentei

## ﻿Additional information

### Author ORCIDs

Ivan L. F. Magalhaes https://orcid.org/0000-0003-3728-3270

Hernán A. Iuri https://orcid.org/0000-0002-8094-9157

Antonio D. Brescovit https://orcid.org/0000-0002-1511-5324

Jaime Pizarro-Araya https://orcid.org/0000-0002-1595-6924

### Data availability

All of the data that support the findings of this study are available in the main text.
